# Rationalizing the relative abundances of trimetallic nitride template-based endohedral metallofullerenes from aromaticity measures†
†Electronic supplementary information (ESI) available: Description of the computational methods used, Fig. S1 with Sc_3_N ALA_N_ analysis, and Fig. S2 and S3 with Y_3_N EMF ALA_N_ analysis. See DOI: 10.1039/c7cc01750b



**DOI:** 10.1039/c7cc01750b

**Published:** 2017-03-21

**Authors:** M. Garcia-Borràs, S. Osuna, J. M. Luis, M. Solà

**Affiliations:** a Department of Chemistry and Biochemistry , University of California , Los Angeles , California 90095 , USA . Email: marcgbq@gmail.com; b Institut de Química Computacional i Catàlisi (IQCC) and Departament de Química , Universitat de Girona , Campus Montilivi , 17003 Girona , Catalonia , Spain . Email: miquel.sola@udg.edu

## Abstract

The synthesis of endohedral metallofullerenes (EMFs) from a carbon soot sample of an arc discharge leads to a variety of EMFs that are obtained in different relative abundances. In the present work, we show that these abundances can be predicted from aromaticity calculations. In particular, we use the normalized Additive Local Aromaticity (ALA_N_) index. Our results show that the most abundant Sc_3_N-based and Y_3_N-based EMFs in fullerene soot are the most aromatic. This study reinforces the idea that aromaticity plays a key role in determining the stability of EMFs.

## Abstract 2

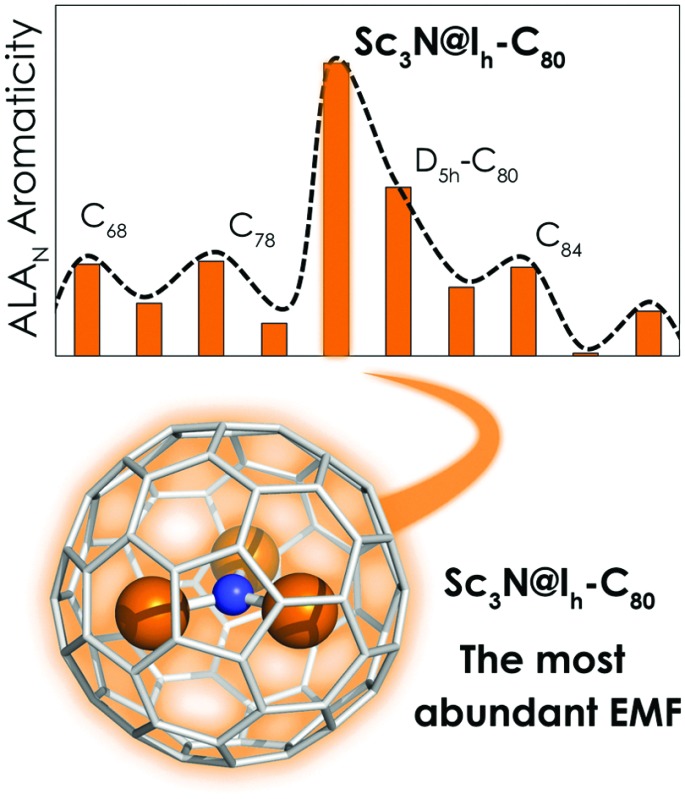
The synthesis of endohedral metallofullerenes (EMFs) from a carbon soot sample leads to a variety of EMFs that are obtained in different relative abundances. In this work, we show that these abundances can be predicted from aromaticity calculations.

Endohedral metallofullerenes (EMFs) have attracted much attention since their discovery because of their extraordinary properties and potential applications.^1–3^ Many different EMFs are obtained by arc discharge or laser vaporization, but only a few of them are produced in macroscopic amounts. The first endohedral metallofullerene obtained in macroscopic quantities was the Sc_3_N@*I*_h_-C_80_ trimetallic nitride template (TNT) EMF, which is the third most abundant fullerene only after C_60_ and C_70_.^4^ The electronic structure of EMFs can be described by the ionic model, as proposed by Poblet and coworkers.^5,6^ When a metallic cluster is encapsulated inside a fullerene cage, a formal charge transfer takes place from the inner moiety to the carbon structure. In the case of TNT moieties, a formal transfer of six electrons takes place; thus its electronic structure can be described as M_3_N^6+^@C_2*n*_^6–^. In 2007, Popov and Dunsch showed that hexaanionic empty fullerene isomers are good models for describing TNT EMF relative stabilities.^7^ Rodríguez-Fortea *et al.* demonstrated that the negative charge is mainly accumulated in the 5-membered rings (5-MRs) of the carbon structure, and that their distribution along the fullerene surface is crucial for stabilizing the final EMF.^8,9^ In 2015, Martín and coworkers showed that the π-energies obtained from a Hückel molecular orbital (HMO) model are useful to predict the stability of anionic and cationic fullerene isomers.^10^ The authors added to the calculated HMO energies an empirical correction of strain energy due to the presence of adjacent pentagon pairs (APPs). Very recently, they demonstrated that the relative isomer stability of anionic fullerenes can be predicted by counting the numbers of three types of hexagon-based motifs and the number of APPs.^11^

A more stable π-electronic structure is likely to be connected with a higher aromaticity. Indeed, it is well-known that the accumulation of negative charge in the 5-MRs of EMFs increases their local and global aromatic character.^12^ In 2013, with the goal of understanding the chemical origin of the stability of anionic fullerene cages, we reported an extensive study showing that, for a given C_2*n*_^–*m*^ cage, the most stable isomers are the ones whose total aromaticity is maximized, independently of the number of APPs they have.^13,14^ We formulated the maximum aromaticity criterion that gave an explanation for the isolated pentagon rule (IPR) violation in EMFs. In this study, aromaticity measurements were carried out in terms of the Additive Local Aromaticity (ALA) index.^13,14^ We used the HOMA geometric descriptor of aromaticity to reduce the computational cost. Still, the HOMA-based calculations provided aromaticity trends similar to those obtained with more reliable, but computationally more expensive electronic multicenter indices.^15^

Studies of EMF stabilities performed to date analyze the relative stability of isomers of a given C_2*n*_^–*m*^ cage. Herein, we have generalized the ALA index to expand its scope and allow the direct comparison of C_2*n*_^–*m*^ cages of different sizes. Our aim is to understand the differences in relative abundances in the production of EMFs by arc discharge of metal/graphite rods. To achieve this goal, we propose the normalization of the ALA index per number of rings present in the fullerene structure (ALA_N_). Using ALA_N_ one can compare the aromaticities of different fullerene cages regardless of the number of carbons of the fullerene structure (see eqn (1)).1
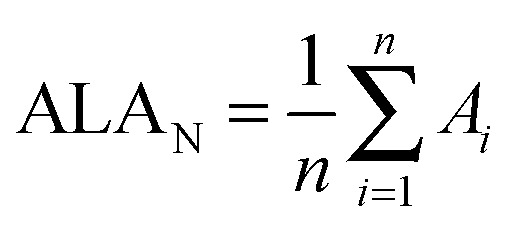
where *A*_*i*_ is the local aromaticity of ring *i*, and *n* is the number of rings of the fullerene structure, including both 5- and 6-MRs. In the present work, the *A*_*i*_ local aromaticities in the ALA_N_ index are computed using the accurate normalized multicenter index, *I*_NB_.^16^ *I*_NB_ allows directly comparing the aromaticities of rings with different sizes. Moreover, we assume that, when calculating the global aromaticity of the fullerene with ALA_N_, all rings in the carbon cage have the same weight. Calculation of the *I*_NB_ electronic index^16–18^ for selected C_2*n*_^6–^ isomers (see the Fig. 1 caption) was performed using the ESI-3D program^19^ at the B3LYP/6-31G//BP86/DZP level of theory, using the Becke's multicenter integration scheme and the topological fuzzy Voronoi (TFVC)^20^ atomic partition scheme as implemented in the APOST-3D program^21^ (see the ESI† for complete computational details). The results obtained are summarized in Fig. 1, where the computed C_2*n*_^6–^ fullerene isomers sorted by their ALA_N_ values are represented. The aromaticities of the C_2*n*_^6–^ cages are compared irrespective of their size and number of APPs.

**Fig. 1:**
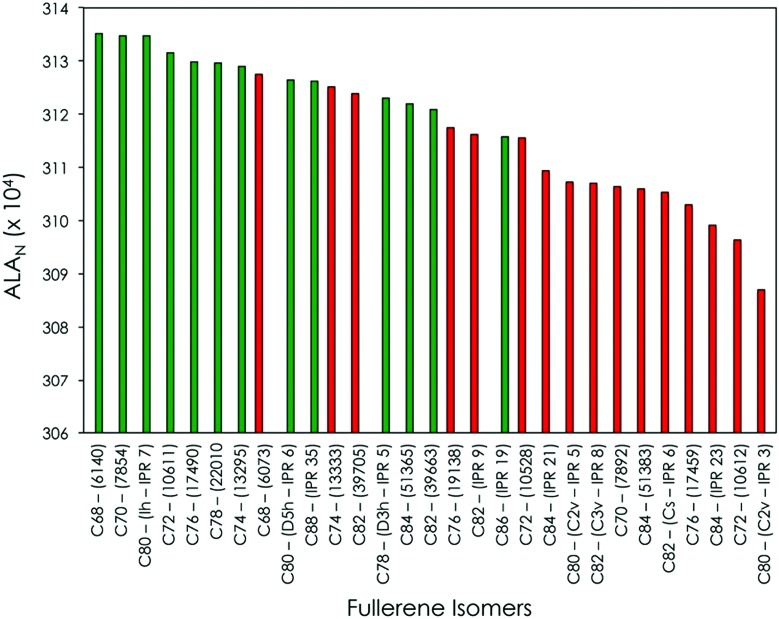
Classification in terms of the ALA_N_ index of selected C_2*n*_^6–^ fullerene isomers. The selection was made including the ALA top-ranked C_2*n*_^6–^ fullerene isomers for each 2*n* (see ref. 13): isomers experimentally detected encapsulating TNTs or M_2_ clusters that formally transfer 6 electrons to the fullerene structure as well as some isomers experimentally detected encapsulating X^4+^ metallic clusters, such as metallic carbides. Isomers experimentally observed forming TNT EMFs are displayed in green, otherwise in red. ALA_N_ values are given in the ALA_N_ × 10^4^ format.

All experimentally detected TNT-containing fullerenes exhibit the largest ALA_N_ values (on the left in Fig. 1 and represented in green), while those hexaanionic isomers that do not form TNT-based EMFs exhibit lower ALA_N_ values (on the right in Fig. 1 and highlighted in red). It is important to mention that, in the present analysis, we consider only the three most stable (and aromatic) isomers characterized in our previous study for each C_2*n*_^6–^ fullerene family (see ref. 13). The rest of the possible isomers present lower ALA_N_ values and have not been experimentally observed as TNT-based EMFs. As a general trend, experimentally observed C_2*n*_^6–^ fullerene isomers containing TNT moieties have the largest ALA_N_ values, thus being the most aromatic ones in terms of the ALA_N_ index. The unique exception to this rule is the isomer C_82_-(39705), which has an ALA_N_ index slightly larger than the experimentally observed TNT isomer M_3_N@C_82_-(39663) (M = Gd and Y). Nevertheless, C_82_-(39663) is the second most aromatic C_82_ hexaanionic isomer, with an ALA_N_ higher than all the other IPR and non-IPR C_82_^6–^ isomers. Our calculations also show that, among cages bigger than C_80_, C_88_-(IPR 5)^6–^ is the most aromatic fullerene (see Fig. 1). Indeed, this is the preferred cage by more bulky 4f-block TNTs such as Ce_3_M, Pr_3_N or Nd_3_N. We can, therefore, conclude that the most aromatic C_2*n*_^6–^ (2*n* = 68–88) fullerene cages, independently of their size or number of APPs, are those most suitable for stabilizing a TNT-based EMF. However, for large TNT moieties like Ce_3_M, Pr_3_N or Nd_3_N, predictions cannot be based only on ALA_N_ studies because steric effects play also an important role.

Encouraged by the promising results obtained from the newly proposed ALA_N_ index using the C_2*n*_^6–^ model, we applied this tool for analyzing the stabilities of Sc_3_N@C_2*n*_ (2*n* = 68–88) EMFs. We computed the ALA_N_ for 10 Sc_3_N-based EMFs explicitly containing the scandium metallic cluster: (6140)-C_68_; (7854)-C_70_; (2210)-C_78_ (Y, Gd, Dy, Tm); (*D*_3h_-IPR 5)-C_78_; (*I*_h_-IPR 7)-C_80_; (*D*_5h_-IPR 6)-C_80_; (39633)-C_82_ (Y, Gd); (51365)-C_84_ (Y, Gd, Tb); (*D*_3_-IPR 19)-C_86_ (Y, Gd, Tb); and (*D*_2_-IPR 35)-C_88_ (Y, Gd, Tb, Lu). In those cases where the Sc_3_N-based EMF has not been experimentally reported, we indicate in parentheses the metals of whose TNTs have been experimentally synthesized. The equivalent ALA_N_ analysis for Y_3_N-based TNT EMFs (C_78_–C_88_) is reported in the ESI.†

In Fig. 2, the relative aromaticities of scandium-based EMFs in terms of ALA_N_ are reported. The first clear conclusion is that Sc_3_N@*I*_h_-C_80_, the most abundant EMF, exhibits the largest normalized aromaticity. Thus, this result suggests a direct relationship between the high stability of this compound and its high aromaticity as compared to other Sc_3_N-based EMFs. More importantly, the ALA_N_-based aromaticity tendencies nicely reproduce the relative abundances of Sc_3_N EMFs observed in the HPLC chromatogram directly obtained from a carbon soot sample of an arc discharge EMF synthesis. The second most abundant and thus presumably second most stable scandium TNT EMF is the *D*_5h_-C_80_. Then, C_68_ and C_78_ fullerenes are the third and fourth most abundant cages. An excellent agreement between the computed relative aromaticities and the relative abundances experimentally observed is therefore found. Our ALA_N_ calculations, in agreement with the experimental HLPC measured relative abundances, also show that the Y_3_N@*I*_h_-C_80_ isomer corresponds to the most abundant Y-based TNT EMFs (see Fig. S2–S4, ESI†). Consequently, we propose that the aromaticity is the chemical driving force that determines the relative stability of the TNT-based EMFs, and we suggest that ALA_N_ measures can be used to predict the relative abundances of still non-synthesized EMFs.

**Fig. 2:**
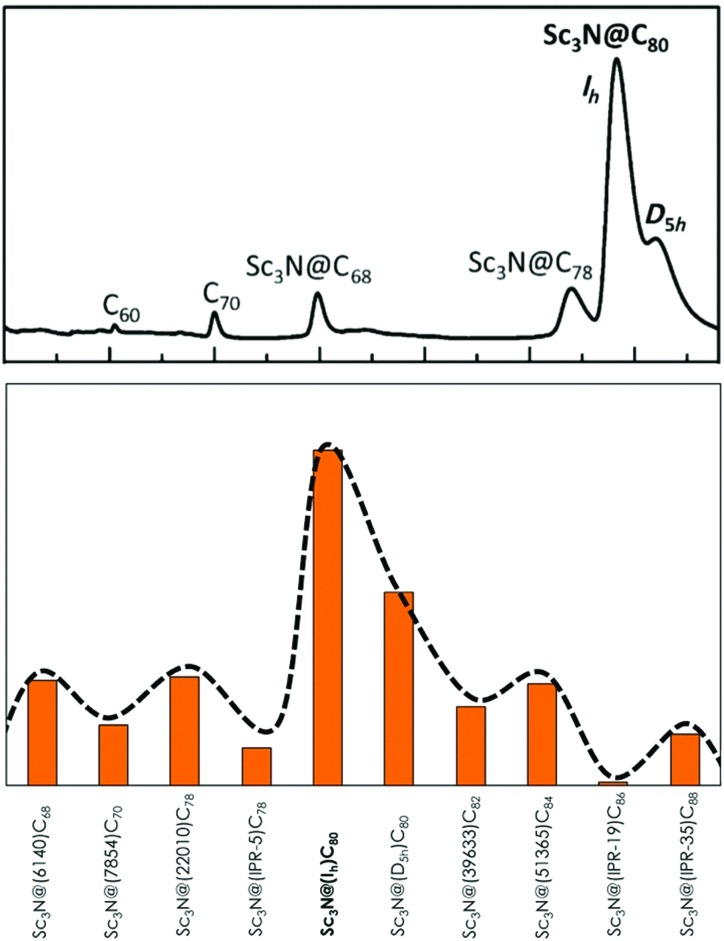
Top: Quantitative HLPC chromatograms of the arc reactor carbon sample of a typical scandium based TNT EMF synthesis (adapted with permission from a previous study by Echegoyen *et al.*, ref. 22). Bottom: Relative ALA_N_ scaled values ((ALA_N_ – ALA_N_(min))/(ALA_N_(max) – ALA_N_(min))), being 1 for Sc_3_N@*I*_h_-C_80_ and 0 for Sc_3_N@(IPR-19)-C_86_, of Sc_3_N@C_2*n*_ fullerenes, sorted by increasing cage size. See the ESI† for equivalent ALA_N_ value graphs.

Minor discrepancies between experimental observations and ALA_N_ predictions are found. For instance, the non-IPR (22010)-C_78_ scandium TNT EMF isomer is slightly more aromatic than the IPR *D*_3h_-C_78_ isomer, despite the IPR is the only experimentally observed as Sc_3_N@C_78_. The non-IPR isomer is, however, the preferred cage for encapsulating large metals such as yttrium or gadolinium.^23^ Our calculations for Y_3_N-based TNTs clearly show the Y_3_N preference for a non-IPR (22010)-C_78_ cage over an IPR *D*_3h_-C_78_ isomer. It should be noted that our analysis does not include thermal contributions that have been shown to be necessary to explain the formation and relative abundances of a given isomer at high temperatures.^24^ In addition, neither kinetic or mechanistic features nor strain effects on the TNT cluster or C_2*n*_ cage are included in our predictions. These factors could explain the minor differences between the theoretically predicted EMF ratios based on their ALA_N_ indices and those experimentally observed in an arc discharge synthesis. Nevertheless, the experimental tendency pointing to the major formation of Sc_3_N-based TNTs *I*_h_-C_80_ and *D*_5h_-C_80_, followed by C_68_ and C_78_, is perfectly reproduced. This result indicates that aromaticity measures in terms of the ALA_N_ index can be used not only to rationalize but also to predict the relative stability (in terms of abundance) of TNT EMFs.

In summary, we proposed a normalized Additive Local Aromaticity index as a measure for the relative stability of any hexaanionic fullerene C_2*n*_^–6^ isomer regardless of their size (from 2*n* = 68 to 88, a typical range for TNT EMF formation), or their number of pentagon adjacencies. We have applied our new index (ALA_N_) to study the relative stabilities of a series of Sc_3_N-based EMFs. Our results show that Sc_3_N@*I*_h_-C_80_, the most abundant EMF, is also the most aromatic, indicating that aromaticity plays a key role in determining its large stability. Even more, the good agreement between the ALA_N_ values obtained for the Sc_3_N- and Y_3_N-based EMF series and the experimental formation ratio indicates that this new measure can be used to understand the chemical origin of the relative stabilities of EMFs, but most importantly to predict their relative abundances.

We are grateful for financial support from the Spanish MINECO (CTQ2014-54306-P, CTQ2014-52525-P, CTQ2014-59212-P, and RyC contract to S.O.), the Catalan DIUE (2014SGR931, ICREA Academia 2014 Award to M.S. and XRQTC), and the FEDER fund (UNGI10-4E-801). M.G.-B. thanks the Spanish MECD for a PhD grant (AP2010-2517) and the Ramón Areces Foundation for a postdoctoral fellowship. S.O. thanks the European Community for the CIG project (PCIG14-GA-2013-630978), and acknowledges the funding from the European Research Council (ERC) under the European Union's Horizon 2020 research and innovation program (ERC-2015-StG-679001). CSUC and BSC-CNS are acknowledged.
