# Supplementary material for: Rationalizing the relative abundances of trimetallic nitride template-based endohedral metallofullerenes from aromaticity measures
Source: Chem Commun (Camb). 2017 Mar 21;53(29):4140–3. doi: 10.1039/c7cc01750b (PMC5436040; doi:10.1039/c7cc01750b)
Supplement: Supplementary file 1 [file CC-053-C7CC01750B-s001.pdf]

Electronic Supplementary Material (ESI) for ChemComm.
This journal is © The Royal Society of Chemistry 2017

SUPPORTING INFORMATION

Understanding the Relative Abundances of TNT-based Endohedral Metallofullerenes from Aromaticity Measures

Marc Garcia-Borràs,[a,b,*] Sílvia Osuna,[b] Josep M. Luis,[b] Miquel Solà[b,*]

a) Department of Chemistry and Biochemistry, University of California, Los Angeles, California 90095, United States

b) Institut de Química Computacional i Catàlisi (IQCC) and Departament de Química, Universitat de Girona, Campus Montilivi, 17003 Girona, Catalonia, Spain.

Computational Details

All Density Functional Theory (DFT) calculations were performed with the Amsterdam Density Functional (ADF) program.[1] The molecular orbitals (MOs) were expanded in an uncontracted set of Slater type orbitals (STOs) of double-ζ (DZP) quality containing diffuse functions and one set of polarization functions. In order to reduce the computational time needed to carry out the calculations, the frozen core approximation has been used.[2] In this approximation, the core density is obtained and included explicitly, albeit with core orbitals (*1s* for C and N, *1s2s2p* for Sc, and *1s2s2p3s3p4s3d* for Y) frozen during the SCF procedure. It was shown that the frozen core approximation has a negligible effect on the optimized equilibrium geometries.[3] Scalar relativistic corrections have been included self-consistently using the Zeroth Order Regular Approximation (ZORA).[4] Energies and gradients were calculated using the local density approximation (Slater exchange) with non-local corrections for exchange (Becke88)[5] and correlation (Perdew86)[6] included self-consistently (i.e. the BP86 functional). The actual geometry optimizations and transition state (TS) searches were performed with the QUILD[7] (QUantum-regions Interconnected by Local Descriptions) program, which functions as a wrapper around the ADF program. The QUILD program constructs all input files for ADF, runs ADF, and collects all data; ADF is used only for the generation of the energy and gradients.[8]

B3LYP/6-31G//BP86/DZP level of theory, using the Becke's multicenter integration scheme and the topological fuzzy Voronoi (TFVC)[9] atomic partition scheme as implemented in APOST-3D program,[10] and ESI-3D program[11] were used to compute the aromaticity electronic normalized multicenter index, I_{NB} index.[12-14] I_{NB} is a normalized version of multicenter index (MCI),[15,16] which is a particular extension of the I_{ring} index.[17]

$$I_{ring}(A) = 2^N \sum_{i_1,i_2,\ldots,i_N}^{OCC} S_{i_1i_2}(A_1)S_{i_2i_3}(A_2)\ldots S_{i_Ni_1}(A_N) \quad (1)$$

being $S_{ij}(A)$ the overlap between molecular orbitals *i* and *j* in the atom A. The MCI index[16] is defined as the sum of all I_{ring} values resulting from the permutations of indices $A_1, A_2, \ldots, A_N$:

$$MCI(A) = \frac{1}{2N}\sum_{P(A)} I_{ring}(A) = \frac{1}{2N}\sum_{P(A)}\sum_{i_1,i_2,\ldots,i_N}^{OCC} S_{i_1i_2}(A_1)S_{i_2i_3}(A_2)\ldots S_{i_Ni_1}(A_N) \quad (2)$$

where *P(A)* stands for a permutation operator which interchanges the atomic labels $A_1, A_2, \ldots, A_N$ to generate the *n*! permutations of the elements in the string *A*.[16] As a result of the large amount of permutations that needs to be computed, this index has associated a high computational cost that limits its use to rings up to nine members. The normalized version of MCI, the I_{NB} index was proposed by Cioslowski, Matito and Solà in 2007.[14]

$$I_{NB}(A) = \frac{C}{NN_\pi}[2N \cdot MCI(A)]^{1/N} \quad (3)$$

where N is the total number of atoms in the ring, N_π is the total number of π electrons, and C ≈ 1.5155, which is a proportionality constant.

Figure S1. On top: Quantitative HLPC chromatograms of the arc reactor carbon sample of a typical scandium based TNT EMFs synthesis (from Echegoyen's Lab, ref. 18); Bottom: ALA_N values (in e^-), sorted by increasing cage size.

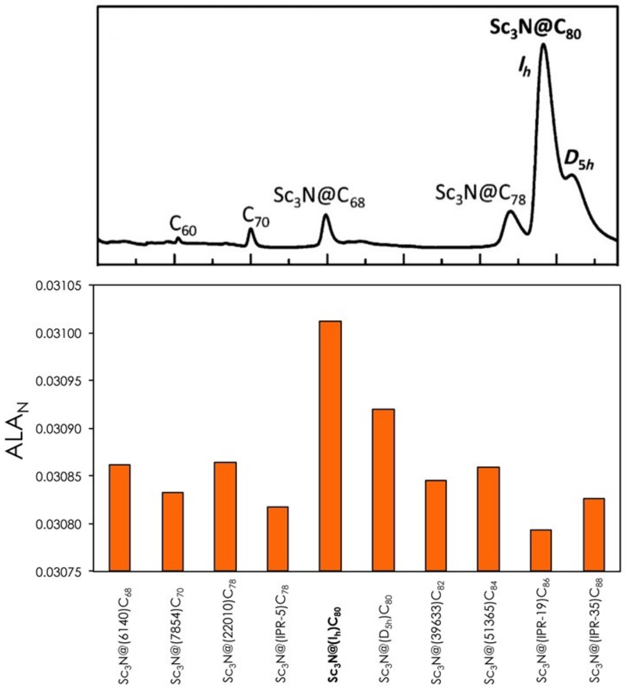

Figure S2. ALA_N analysis of Y_3N EMFs relative abundances at B3LYP/6-31G+Y(SDD)//BP86/DZP level of theory

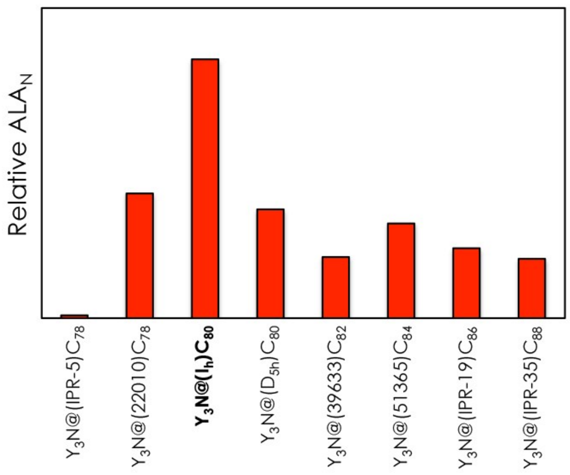

$Y_3N@$	ALA_N x 10^4	Relative ALA_N
(D_{3h})-C_{78}	308.26	1
(22010)-C_{78}	309.39	48
(I_h)-C_{80}	310.60	100
(D_{5h})-C_{80}	309.24	42
(39633)-C_{82}	308.82	24
(51365)-C_{84}	309.12	37
(IPR-19)-C_{86}	308.89	27
(IPR-35)-C_{88}	308.80	23

Figure S3. Quantitative HPLC chromatograms of the arc reactor carbon sample of a yttrium based TNT EMFs synthesis from J. Am. Chem. Soc. 2009, 131, 11762.

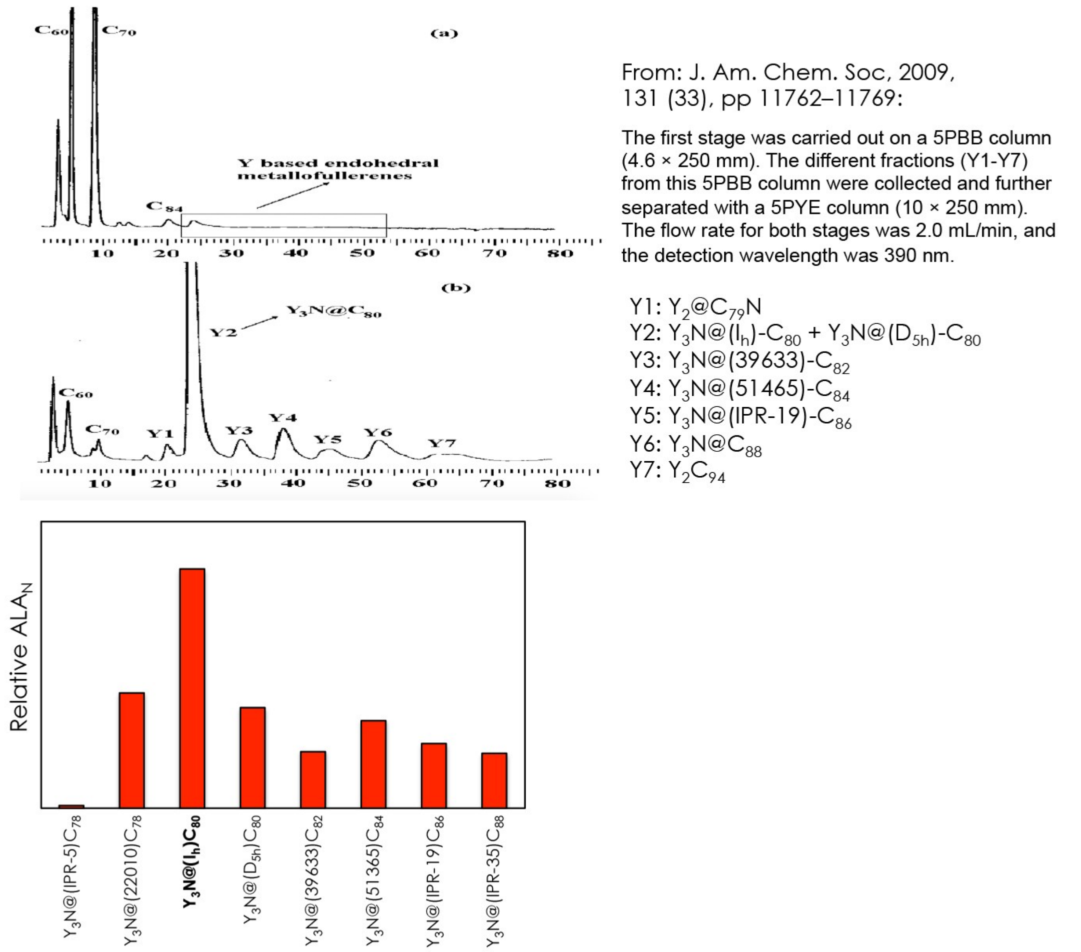

$Y_3N@(I_h)$-C_{80} + $Y_3N@(D_{5h})$-C_{80} peak corresponds to the most abundant mixture of Y-based TNT EMFs, in agreement with ALA_N predicted abundances. Also, relative abundances of C_{82}-C_{86} Y_3N EMFs are correctly predicted by ALA_N measures.

Nevertheless, Dorn and co-workers were not able to isolate and properly characterize the Y6 peak supposed to be $Y_3N@(IPR$-$35)$-C_{80} (J. Am. Chem. Soc. 2009, 131, 11762), thus not ensuring its purity.

Figure S4. Quantitative HPLC chromatograms of the arc reactor carbon sample of a yttrium based TNT EMFs synthesis from J Nanoscale, 2011, 3, 4955.

From: Nanoscale, 2011, 3, 4955

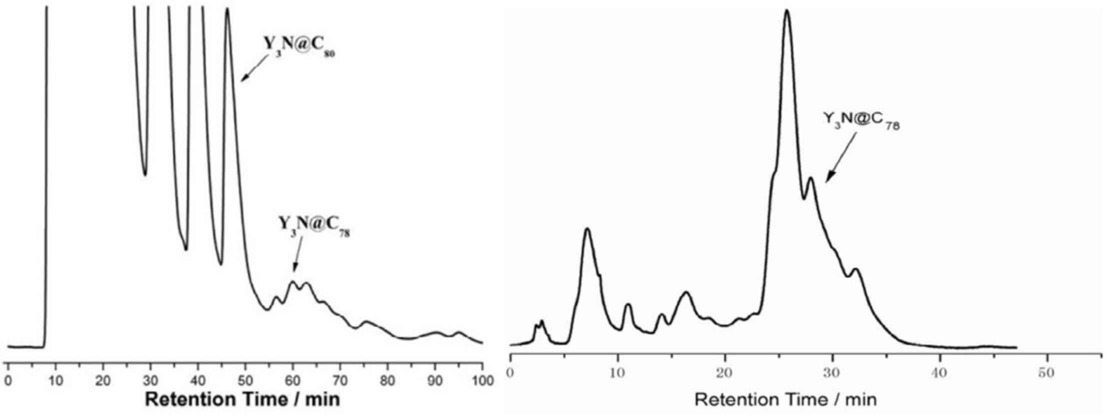

The first stage HPLC profile of toluene extract of the soot containing mostly yttrium endohedral metallofullerenes (20×250 mm Buckyprep column; flow rate 12 mL/min; toluene as eluent).

The second stage HPLC profile of Y3N@C78 fullerenes (20×250 mm Buckyprep-M column; flow rate 12 mL/min; toluene as eluent).

From "Nanoscale, 2011, 3, 4955":

"*The sample was synthesized by the Kräschmer–Huffman arc discharging method and purified by two-stage high performance liquid chromatography (HPLC). Two complementary columns, i.e. Cosmosil Buckyprep and Buckyprep-M, were employed to isolate and purify the $Y_3N@C_{78}$. The $Y_3N@C_{78}$ was detected followed by $Y_3N@C_{80}$ on Buckyprep column, whereas the $Sc_3N@C_{78}$ has a shorter retention time than $Sc_3N@C_{80}$. Such abnormality has indicated how special this target molecule is.*"

The first isolation and HPLC characterization of Y_3N@(22010)-C_{78} EMF by Ma et al. (Nanoscale, 2011, 3, 4955) showed the particularities of this EMF, and the reasons why Dorn and coworkers were not able to purify by HPLC this compound (J. Am. Chem. Soc. 2009, 131, 11762).

References:

(1) Baerends, E. J. et al. ADF2010, Amsterdam, 2010.
(2) te Velde, G.; Bickelhaupt, F. M.; Baerends, E. J.; Fonseca Guerra, C.; van Gisbergen, S. J. A.; Snijders, J. G.; Ziegler, T. *J. Comput. Chem.* **2001**, *22*, 931.
(3) Swart, M.; Snijders, J. G. *Theor. Chem. Acc.* **2003**, *110*, 34.
(4) van Lenthe, E.; Baerends, E. J.; Snijders, J. G. *J. Chem. Phys.* **1993**, *99*, 4597.
(5) Becke, A. D. *Phys. Rev. A* **1988**, *38*, 3098.
(6) Perdew, J. P. *Phys. Rev. B* **1986**, *33*, 8822.
(7) Swart, M.; Bickelhaupt, F. M. *J. Comput. Chem.* **2008**, *29*, 724.
(8) Swart, M.; Bickelhaupt, F. M. *Int. J. Quant. Chem.* **2006**, *106*, 2536.
(9) Salvador, P.; Ramos-Cordoba, E. *J. Chem. Phys.* **2013**, *139*, 071103.
(10) Salvador, P.; Ramos-Cordoba, E. APOST-3D, Institut de Química Computacional i Catàlisi, Universitat de Girona, Girona, 2011.
(11) Matito, E. ESI-3D: Electron sharing indexes program for 3D molecular space partitioning, Institut de Química Computacional i Catàlisi, Universitat de Girona, Girona, 2006.
(12) Matito, E.; Duran, M.; Sola, M. *J. Chem. Phys.* **2005**, *122*, 014109.
(13) Matito, E.; Solà, M.; Salvador, P.; Duran, M. *Faraday Discuss.* **2007**, *135*, 325.
(14) Cioslowski, J.; Matito, E.; Sola, M. *J. Phys. Chem. A* **2007**, *111*, 6521.
(15) Bultinck, P.; Fias, S.; Ponec, R. *Chem. Eur. J.* **2006**, *12*, 8813
(16) Bultinck, P.; Ponec, R.; Van Damme, S. *J. Phys. Org. Chem.* **2005**, *18*, 706.

(17) Giambiagi, M.; de Giambiagi, M. S.; dos Santos, C. D.; de Figueiredo, A. P. *Phys. Chem. Chem. Phys.* **2000**, *2*, 3381.
(18) Cerón, M. R.; Li, F. F.; Echegoyen, L. *Chem. Eur. J.* **2013**, *19*, 7410.
